# Shifting drug markets in North America - a global crisis in the making?

**DOI:** 10.1186/s13033-023-00601-x

**Published:** 2023-10-25

**Authors:** Maximilian Meyer, Jean N. Westenberg, Kerry L. Jang, Fiona Choi, Stefanie Schreiter, Nickie Mathew, Conor King, Undine E. Lang, Marc Vogel, R. Michael Krausz

**Affiliations:** 1 Department of Psychiatry, Faculty of Medicine, University of British Columbia, 5950 University Boulevard, Vancouver, BC V6T 1Z3 Canada; 2 University Psychiatric Clinics Basel, University of Basel, Basel, Switzerland; 3 Department of Psychiatry and Neurosciences, Charité – Universitätsmedizin Berlin, Berlin, Germany; 4 Victoria Police Department, Victoria, Canada

**Keywords:** Drug market, Opioid overdose crisis, Fentanyl, Opioid use disorder, Market development

## Abstract

Understanding drug market dynamics and their underlying driving factors is paramount to developing effective responses to the overdose crisis in North America. This paper summarises the distinct drug market trends observed locally and internationally over the past decade to extrapolate future drug market trajectories. The emergence of fentanyl on North American street markets from 2014 onwards led to a shift of street drug use patterns. Previously perceived as contaminants, novel synthetic opioids became the drugs of choice and a trend towards higher potency was observed across various substance classes. The diversification of distribution strategies as well as the regionalisation and industrialisation of production followed basic economic principles that were heavily influenced by prosecution and policy makers. Particularly, the trend towards higher potency is likely most indicative of what to expect from future illicit drug market developments. Nitazenes and fentanyl-analogues, several times more potent than fentanyl itself, are increasingly detected in toxicological testing and have the potential of becoming the drugs of choice in the future. The dynamic of drug import and local production is less clear and influenced by a multitude of factors like precursor availability, know-how, infrastructure, and the success of local drug enforcement strategies. Drug market dynamics and the current trajectory towards ultrapotent opioids need to be recognised by legislation, enforcement, and the health care system to prepare effective responses. Without significant improvements in treatment access, the implementation of preventative approaches and early warning systems, the mortality rate will continue to increase. Furthermore, there is no mechanism in place preventing the currently North American focused overdose crisis to spread to other parts of the globe, particularly Europe. A system of oversight, research, and treatment is needed to address mortality rates of historic proportions and prevent further harm.

## Introduction

The overdose crisis death toll in the United States (US) exceeds 100,000 fatalities each year and represents the highest mortality due to a mental health condition [1, 2]. In recent years, heroin, the formerly dominant street opioid in North America, has been systematically replaced by fentanyl [3]. This synthetic opioid is approximately 70 times more potent than morphine and has become a main driver of the overdose crisis [4]. Extremely high levels of prescription opioid use in the early 2000’s have contributed to the crisis [5]. However, countries like Germany have similarly high rates of opioid prescriptions and yet, are not experiencing a shift from heroin to fentanyl, nor are they experiencing an increase in overdose deaths. This suggests a much more intricate array of factors that can account for the ongoing development in North America [6]. Indeed, it is much more likely that a complex entanglement of economic and structural factors resulted in the unprecedented drug market shift towards synthetic opioids. This shift changed nearly everything that has been assumed so far regarding drug markets, patterns of use, and drugs of choice.

We aim to lay out the most important drug market trends of the past decade, project the most likely future developments, and discuss potential responses from the system of care. Studying these changes might allow a better inference as to what will be the most likely upcoming trajectory of the illicit synthetic opioid supply, its impact on opioid use, and the consequences for the system of care. These predictions can have tremendous implications as they allow the health care system, drug enforcement, and the legislative system to prepare the necessary efficacious responses to slow-down and reverse the present rising fatality rates.

### Major drug market trends of the past decade

Drug markets are not homogenous and differ considerably across different regions, cultures, and substance classes. These specifics might result from differences in enforcement strategies, the legal framework, the approach of drug cartels and producers, as well as the functionality of the health care system. The following trends can be observed internationally as well as in the different segments of local North American drug markets.

#### Synthetic opioids as drug of choice

From 2014 onwards, there was an emergence of highly potent synthetic opioids, specifically fentanyl, on the street drug markets in North America. What started out as a traditional heroin market with occasional fentanyl appearances, grew to a widespread availability through the contamination of street heroin with imported fentanyl. This later shifted towards locally produced fentanyl due to several factors, including its economic advantages and a crack-down on fentanyl in China [7]. Indeed, in 2019, China started controlling fentanyl by criminalising its production and export, which undoubtedly further pressured an already growing local production of fentanyl [8]. Once fentanyl had saturated the North American drug markets, it was not long before fentanyl became the opioid of choice for a proportion of individuals who use opioids [9–11]. Concurrently, “pure” heroin (i.e., heroin that is not contaminated with synthetic opioids) became rare in some metropolitan areas in North America. Meanwhile, seizures of heroin have remained stable over the past decade in the European Union, with the amount of seized fentanyl substantially increasing only in Lithuania [12].

#### The trend of higher potency

The current trend towards the use of high potent synthetic opioids, especially fentanyl, has caused an extreme increase in the prevalence of non-fatal and fatal overdose events [13, 14]. However, the trend towards higher opioid potency is still ongoing, with several analogues and novel opioids becoming increasingly available. Particularly carfentanil, an analgesic used in veterinary medicine to anesthetise elephants, provides reason for concern. It is estimated to be around 10’000 times more potent than morphine. In 2021, 8% of all illicit drug toxicity deaths in British Columbia (BC) Canada involved this agent [14, 15]. Furthermore, benzimidazole opioids or “nitazenes” emerged in North America and have since gained a foothold in its drug street markets [16, 17]. Nitazenes and their analogues can exceed the potency of fentanyl by a factor of ten and their availability is steadily increasing [18]. For example, isotonitazene was identified in Canadian drug seizures 12 times in 2019, a number that increased to 288 times in 2021 [19].

#### Ongoing chemical modification of novel psychoactive substances

Novel synthetic opioids like nitazenes are also the subject of rapid structural development and challenge forensic chemists in their efforts to keep up with toxicological profiling and characterisation [20]. The trend of rapid chemical modification also extends to cannabinoids and stimulants, requiring policy makers to constantly amend their lists of controlled substances [21–23]. The use of these novel modified psychotropic substances has been associated with fatal outcomes and numerous, sometimes life-threatening, side effects which are reported in literature [24–26]. In addition to the severe risk associated with the use of these substances, they represent ever-moving targets that complicate the legislative as well as health care response.

#### Regionalisation of production based on precursors

Aggravated by China’s controlling efforts, fentanyl production in North America is to date mainly happening regionally and based on smuggled precursors [27]. In the US, the largest share of fentanyl is produced in and imported from Mexico, whereas repeated dismantling of drug laboratories in Canada suggest regionalised production within country borders [28–30]. By reducing the risk that comes with the trading and smuggling of opioids over long distances and across multiple country borders, drug dealers are able to increase the availability of their product [3]. Furthermore, domestic production is a key strategy to reduce drug prices as the biggest markups typically occur at the time of border-crossing [31].

#### Industrialisation of production and distribution

Similar to the opioid and cannabinoid market, the stimulant market is subject to change. In recent years, there was a continuous growth in methamphetamine seizures whereas seizures of cocaine declined slightly in the US [32]. The underlying reason for this development is an industrial-style upscale of drug production in Central and South America [9]. Methamphetamine is produced in large quantities in Mexico, with drug cartels expanding and utilising their networks for distribution in North America. This model of production and distribution is continuously gaining traction, even though it demands significantly higher levels of control, larger amounts of resources and sophisticated logistics compared to smaller scale “drug kitchens”. Both can be provided in countries in which drug cartels are able to operate, like Mexico. The resulting high-volume production allows drug market control with lower prices and production costs.

#### Expansion of drug distribution networks: online distribution over the dark web

Distribution strategies and logistics have also diversified in the past years. Distribution of drugs from dealer to dealer but also from dealer to user is increasingly happening online through the darknet [34]. Online distribution comes with the potential advantage of reducing the risk of prosecution for both parties. Darknet markets can be used by local drug dealers and manufacturers to buy precursor substances, but are also attractive for people who use drugs (PWUD) as they offer a comprehensive range of drugs.

#### Growing impact of novel stimulants

The prevalence of stimulant use is increasing, and novel synthetic stimulants enter the market each year. Today, stimulants account for the largest class of novel psychoactive substances discovered and monitored in Europe [22]. In North America and Europe alike, they have commonly been sold legally (e.g., as bath salts) and mis-sold illegally as MDMA [35–37]. The use of these novel stimulants can lead to severe intoxications with psychotic episodes, violent behaviour, days-long insomnia, cardiac arrest, and death [26]. Given their increased availability and relatively low prices, stimulants are particularly harmful due to their popularity among adolescents and young adults.

#### Increase in polydrug use: benzodiazepines and xylazine

Combining opioids with benzodiazepines to increase potency and prolong the duration of effect has become a widespread practice [38]. Initially, fentanyl was combined with heroin and termed “fentanyl with legs”, as heroin prolonged the otherwise quickly subsiding fentanyl rush [3]. However, with heroin becoming rare on North American street markets, the practice shifted towards the combination of fentanyl with benzodiazepines. This poses a problem for first responders as different antagonising agents are required in case of overdose events. Etizolam is currently among the most prevalent “novel” benzodiazepines. Compared to other benzodiazepines, it is short-acting, has a fast onset of effect and is 5–10 times more potent than diazepam [39]. It has become highly sought after in recent years for the purpose of “providing legs”, the practice of mixing it with a short-acting opioid thereby prolonging its duration of effect. The popularity of this practice likely led to etizolam now ranking among the most seized substances in Canada in 2022 [40]. In the coroner’s report from BC, this translated to a steep incline in the presence of benzodiazepines in drug toxicity deaths, with etizolam being involved in 35% of deaths that underwent expedited toxicological testing [14]. Another adulterant, that is increasingly identified in combination with fentanyl, heroin and cocaine and has been linked to a growing number of overdose deaths and injection-related soft tissue damage is xylazine, which has also been found to give fentanyl “legs” [41, 42]. Xylazine is a clonidine-analogue typically used in veterinary anaesthesia. In humans, its side effects include respiratory depression, bradycardia, hypotension, with intoxications often leading to fatal outcomes [43]. From 2020 to 2021 xylazine-positive overdose deaths increased tenfold in the south of the US, with its presence in North America likely being underestimated due to toxicologic testing often not including this agent [41].

#### The impact of prescription opioids

An undisputable contributing factor to this overdose crisis is prescription opioids, which were widely available at the turn of the century and coupled with insufficient regulation of the pharmaceutical and healthcare industries [44]. Strong prescription control was then put in place, but without an expansion of pain and addiction treatment [44]. Among the many unintended consequences of such restrictions on prescribing practices included the growth of illicit drug market with counterfeit pills, heroin, and eventually fentanyl [44]. Although prescription opioid-involved deaths rates have remained relatively stable, it is important to remember what role prescription drug-control policies had in shaping the nature of today’s drug markets [5].

### The future trajectory of north american drug markets

The trend towards higher potency is likely most indicative of what to expect from future illicit substance market developments. According to Canadian drug seizure statistics heroin is no longer among the top ten recovered illegal substances [32, 40]. Fentanyl is currently the most-seized opioid but likely in the process of being replaced with even more potent synthetic opioids [10, 11]. This trend is exemplified by the fact that carfentanil is now being found more frequently than heroin during drug seizures [40]. Fentanyl’s evolutions to the drug of choice demonstrates how the market supply itself can define and change drug use patterns [10]. Although there is currently no clear indication of individuals specifically seeking and prioritising nitazenes or fentanyl-analogues over fentanyl, these substances have the potential of becoming the drug of choice in the near future. The transition to ultra-potent synthetic opioids could resemble the one that happened 5 years ago with fentanyl. That is, individuals who use opioids will be gradually exposed to these drugs, as the fentanyl sold will be cut, knowingly or unknowingly to the individuals using it [11, 45]. The exposure to these substances will likely lead to increased tolerance, and fentanyl alone may no longer provide the sought after physical and psychological effect it once did. This allows agents that are initially perceived to be contaminants to become to the drug of choice. Notably, a shift towards less potent and less dangerous substances has to date not been observed either in opioid markets or in other substance classes, making this development seem unidirectional and irreversible.

The market dynamic of import and local production is of particular interest for legislation and prosecution. However, its future trajectory is less clear. A trend towards ultrapotent opioids would generally mean that less total amount of substance is needed to be smuggled across the border. Therefore, the more potent a substance is, the smaller amount needs to be transported and the lower the risk of seizures for drug traffickers. The logical pathway forward for drug traffickers would hence be a strong preference for the local production and international distribution of highly potent substances. This has previously been referred to as the ‘Iron Law of Prohibition’, whereby pressures due to attempted interruption and suppression of the illicit drug supply leads to ever-more compact substitutes [46]. Following China’s announcement to control fentanyl in 2019, several local drug laboratories emerged in Mexico and other North American metropolitan areas [30]. Concurrently, Mexican methamphetamine imports increased, making the product a cheap and successful competitor to locally produced methamphetamine. According to police reports this resulted in pre-existing Canadian labs shifting their focus towards fentanyl production as well. The Australian police has recently seized a large shipment of fentanyl and methamphetamine from Canada, indicating that the upscale in North America’s local drug production has already resulted in the area becoming a net exporter of drugs [47]. It is possible that the trend of local production of ultrapotent opioids in small laboratories, as currently observed in BC, will spread out to other urban areas in the US, making imports of these substances entirely obsolete. However, this development depends on a multitude of factors like resources and precursor accessibility, drug cartel influence, mechanisms of drug enforcement, as well as the required know-how of drug chemists and the necessary production infrastructure. For example, organised crime groups exercise significant control of the drug trade in some larger US cities and are unlikely to tolerate competition from local producers.

### Starting points for an effective system-level response

Health care systems across the North American continent have so far been unable to effectively respond to the continuously increasing overdose mortality rates [17]. Without significant improvements in the system of care, the implementation of preventative approaches and early warning systems, as well as specialised services for adolescents and young adults, the mortality rate will likely continue to increase [48]. Equally, purely legislative measures like prohibition and prosecution of substance possession and personal use have so far also been unsuccessful in stopping the flourishing drug market and reducing the mortality rate in North America. This is recognised by Canadian legislative bodies and first policy changes are underway: from January 2023, possession and personal use of drugs (up to 2.5 g) are no longer a criminal offense in the province of BC [49]. Instead of seizing the substance, police forces will provide information on health and social services. This law was adopted for a period of three years and will be subject to ongoing evaluation. However, it is unclear how this approach will improve individual health outcomes since current treatment capacities are not being expanded and access barriers to health services will still be in place.

Indeed, the most effective treatment approach for opioid use disorder is opioid agonist treatment (OAT) [50]. Still, in the US, treatment coverage is estimated to be 28% and waiting times for treatment services can extend to several weeks [51]. Furthermore, OAT in North America is usually not individualised, meaning that most patients receive either sublingual buprenorphine or oral methadone [52]. Slow-release oral morphine and fast-acting agonists are utilised in Canada to some extent, but treatment barriers like waiting times and long-distance travel to reach clinics limit access to them [53, 54]. These barriers as well as the limited availability of substances beyond buprenorphine and methadone can discourage PWUD to seek OAT or even lead to potentially harmful self-medicating behaviours. For instance, illicit benzodiazepines may be sought by PWUD to self-manage opioid withdrawal or enhance opioid potency [55]. This mixing of substances can lead to the development of multiple concurrent substance use disorders and even complicate overdose management (i.e., different antagonists are required to treat benzodiazepine and opioid overdoses). It is therefore imperative for psychiatrists, and mental health professionals more broadly, to leverage their professional status to advocate for a diversification of available opioid agonists, given the research indicating their safety and efficacy. In a naturalistic and liberal opioid agonist treatment setting, the evidence demonstrates that there is no excessive demand for a single medication [56]. Rather, all evidence-based treatment options should be added to the armamentarium in the treatment of opioid use disorder. This would allow patients, in conjunction with their healthcare team, to decide which medication suits their needs best, in order to optimise treatment retention and health outcomes.

There are lessons to be learned from European countries like Switzerland and the Netherlands. These countries broke with established paradigms and successfully tackled the heroin- and HIV-crisis of the 1990s by implementing heroin-assisted treatment as a novel and effective treatment regimen [57]. To this day, strategies that directly address the issue of treatment attractiveness like intranasal heroin-assisted treatment and extended take-home doses are actively being investigated and early results are promising [58–61]. These strategies to improve treatment attractiveness are needed in North America and novel approaches like fentanyl-assisted treatment have already been proposed but are far from being evaluated and implemented [62].

### A global overdose crisis in the making?

Drug market dynamics and the current trajectory towards ultrapotent opioids need to be recognised by legislation, enforcement, and the health care system to prepare effective responses. Even though response frameworks for health care systems and policy models from other countries exist, the US and Canada have so far been unable to develop an effective strategy [63]. Past strategies like fentanyl test services and traditional OAT were not successful in stopping the continuously rising mortality. Equally, recently implemented strategies like the “safer supply” of drugs has had no impact on the development so far [64, 65]. What does that mean for the coming years globally? Seizures by the Australian police indicate that North America has started exporting fentanyl to other continents. Until now, these exports had little impact on Australian drug markets which is probably owed to the diligent work of Australian border control forces [66]. However, only limited mechanisms exist to prevent the currently North American focused overdose crisis to spread to other parts of the globe.

## Conclusions

Mental health experts, particularly psychiatrists, need to step up and follow a comprehensive and integrated approach to addressing the opioid overdose crisis. The importance of the mental health care system is rooted in its capacity to address the co-occurrence of mental illness and substance use disorders, provide access to substance use treatment, integrate mental health and substance use services, and understand the psychological and social factors contributing to substance use disorders. Treatment barriers like waitlists and long distances to the nearest treatment provider need to be overcome by increasing treatment capacity and through cooperation and training of family physicians to provide OAT. Advocating for an expanded access to evidence-based medications is an important place to start. An effective response therefore requires the individual efforts of psychiatrists as well as a system change focused around integrating mental health care, social services, and counselling. The current North American system of interventional silos between specialities must be abandoned to be able to respond to future drug market shifts and their potentially detrimental public health impact. All health care professionals must be empowered when treating patients with opioid use disorder, no matter their specialisation. This should include specialty-appropriate education and training for proper referral or management of patients. Such system-wide changes should be orchestrated by psychiatrists, other mental health professionals, and perhaps most importantly, patients and their families.

## Data Availability

Not applicable.

## Abbreviations

US: United States
BC: British Columbia
PWUD: People who use drugs
OAT: Opioid agonist treatment
